# Mitochondrial antioxidant SkQ1 decreases inflammation following hemorrhagic shock by protecting myocardial mitochondria

**DOI:** 10.3389/fphys.2022.1047909

**Published:** 2022-11-16

**Authors:** Bo Jia, Jingjing Ye, Lebin Gan, Rui Li, Mengwei Zhang, Diya Sun, Lin Weng, Yufei Xiong, Jun Xu, Peng Zhang, Wei Huang, Ming Zheng, Tianbing Wang

**Affiliations:** ^1^ Trauma Medicine Center, Peking University People’s Hospital, Key Laboratory of Trauma and Neural Regeneration (Peking University), Ministry of Education, National Center for Trauma Medicine of China, Beijing, China; ^2^ School of Basic Medical Sciences, Department of Physiology and Pathophysiology, Peking University Health Science Center, Beijing, China; ^3^ Department of Gastroenterology, Clinical Center of Immune-Mediated Digestive Diseases, Peking University People’s Hospital, Beijing, China

**Keywords:** hemorrhagic shock, SkQ1, mitochondria, inflammatory response, MTDs

## Abstract

**Background:** Hemorrhagic shock (HS) is a type of hypovolemic shock characterized by hemodynamic instability, tissue hypoperfusion and cellular hypoxia. In pathophysiology, the gradual accumulation of reactive oxygen species (ROS) damages the mitochondria, leading to irreversible cell damage and the release of endogenous damage-associated molecular patterns (DAMPs) including mitochondrial DAMPs (MTDs), eventually triggering the inflammatory response. The novel mitochondria-targeted antioxidant SkQ1 (Visomitin) effectively eliminate excessive intracellular ROS and exhibits anti-inflammatory effects; however, the specific role of SkQ1 in HS has not yet been explicated.

**Methods and results:** A 40% fixed-blood-loss HS rat model was established in this study. Transmission electron microscopy showed that after HS, the myocardial mitochondrial ultrastructure was damaged and the mtDNA release in circulation was increased and the differentially expressed genes were significantly enriched in mitochondrial and ROS-related pathways. Mitochondria-targeted antioxidant SkQ1 attenuated the increased ROS induced by HS in myocardial tissues and by oxygen-glucose deprivation (OGD) in cardiomyocytes. Ultrastructurally, SkQ1 protected the myocardial mitochondrial structure and reduced the release of the peripheral blood mtDNA after HS. RNA-seq transcriptome analysis showed that 56.5% of the inflammation-related genes, which altered after HS, could be significantly reversed after SkQ1 treatment. Moreover, ELISA indicated that SkQ1 significantly reversed the HS-induced increases in the TNF-α, IL-6, and MCP-1 protein levels in rat peripheral blood.

**Conclusion:** HS causes damage to the rat myocardial mitochondrial structure, increases mtDNA release and ROS contents, activates the mitochondrial and ROS-related pathways, and induces systemic inflammatory response. The mitochondrial antioxidant SkQ1 can improve rat myocardial mitochondria ultrastructure, reduce mtDNA and ROS contents, and decrease inflammation by protecting myocardial mitochondria, thereby playing a novel protective role in HS.

## Introduction

Hemorrhagic shock (HS) is a form of hypovolemic shock and is characterized by hemodynamic instability, tissue hypoperfusion, and cellular hypoxia (Peitzman et al., 1995; Cannon, 2018). Annually, 1.9 million deaths occur from hemorrhage worldwide, indicating that it is a severe substantial global problem (Lozano et al., 2012). Moreover, patients who survive the initial hemorrhagic injury exhibit poor functional outcomes and significantly elevated long-term mortality (Mitra et al., 2014; Halmin et al., 2016). In pathophysiology, due to inadequate oxygen delivery at the cellular level, the metabolism changes from aerobic to anaerobic, which leads to the gradual accumulation of lactic acid and reactive oxygen species (ROS) and reduced ATP supply, eventually resulting in cellular dyshomeostasis, irreversible cell damage, and death (Cannon, 2018) HS-induced cellular injury can lead to the release of endogenous damage-associated molecular patterns (DAMPs) including mitochondrial DAMPs (MTDs) into circulation and subsequently activates the innate immune response (Zhang et al., 2010).

Dysfunctional mitochondria are the key sources of DAMPs (Krysko et al., 2011). MTDs is one of the important mechanisms of trauma. It can not only induce the immune response and aggravate the inflammatory response but also lead to tissue and organ damage. A previous study reported that the level of MTDs including formyl peptide and mitochondrial DNA (mtDNA) significantly increased in the tissue and circulatory system of patients with trauma, which activated neutrophils through formyl peptide receptors and led to an increase in the level of intracytoplasmic calcium ions (Zhang et al., 2010; Hotz et al., 2018; Faust et al., 2020). Additionally, MTDs promoted the migration and degranulation of neutrophils and triggered systemic inflammatory damage and sepsis-like response. This finding also partly explains the mechanism of traumatic sepsis without infection (Horn et al., 2022). Moreover, changes in the mtDNA content in blood were positively correlated with the changes in inflammatory indices such as lung MPO and blood IL-6 (Aswani et al., 2018), indicating that plasma mtDNA possibly drives early inflammation after traumatic HS. However, the effect of mitochondria in the heart after HS has not been studied yet.

The antioxidant SkQ1 is the first mitochondrial-targeted drug in clinical application (Yani et al., 2012). SkQ1 is structurally similar to MitoQ, differing only in the antioxidant molecule (ubiquinone is replaced by plastoquinone) (Zhao et al., 2005). Replacing ubiquinone with plastoquinone can promote the antioxidant activity of mitochondrial targeted compounds and increase the interaction among pro-oxidants, antioxidants, and ROS (Skulachev, 2007). SkQ1 at extremely low (nanomolar) concentrations can effectively remove excessive ROS during brain injury, cerebral ischemia, and Alzheimer’s disease, thereby reducing the adverse effects of ROS in these animal models (Plotnikov et al., 2010; Kapay et al., 2011; Isaev et al., 2012; Kirpatovsky et al., 2013; Genrikhs et al., 2015). Nevertheless, the specific role of SkQ1 in HS has not yet been explicated.

In the present study, we established a 40% fixed-blood-loss HS rat model (Chang et al., 2018) and interestingly, we found that 12 h of hemorrhagic shock caused damage to the rat myocardial mitochondrial structure, increased mtDNA release and ROS content, activated the mitochondrial and ROS-related pathways and indeced the systemic inflammatory response. We applied SkQ1 in HS treatment for the first time in this study and found that SkQ1 could reduce inflammation following hemorrhagic shock by protecting myocardial mitochondria.

## Materials and methods

### Rat hemorrhagic shock model

All animal procedures were performed in accordance with the Laboratory Animal Guideline for the Ethical Review of the Animal Welfare of China, and were approved by the Medical Ethics Committee of the Peking University People’s Hospital (2020PHE063). Hemorrhagic shock was induced in the rat model as described previously (Wang et al., 2021). Briefly, male Sprague-Dawley rats (body mass, 240–310 g) were anaesthetised with 1%–3% vaporised isoflurane, followed by sterile placement of femoral vascular by using a polyethylene 50 tube flushed with heparinised saline. The HS group SD rats were subjected to 40% total blood volume hemorrhage [estimated blood loss volume (mL) = mass(g) × 0.06 + 0.77], while the sham group rats were anaesthetised and subjected to femoral cannulation without hemorrhage. The rats were euthanised 12 h after hemorrhage for harvesting serum and tissue samples. After establishing the rat HS model, the rats in the HS + SkQ1 group were intraperitoneally injected with SkQ1 (1 μM/kg) immediately after HS surgery, whereas those in the HS + vehicle group were injected with the same dose of vehicle. The optimal dose for intraperitoneal injection of SkQ1 (1 μM/kg) was reported in the previous study (Bakeeva et al., 2008).

### Isolation and culture of neonatal rat cardiomyocytes

Neonatal rat cardiomyocytes were isolated and cultured, as previously described (Ye et al., 2018). The ventricular myocytes of Sprague-Dawley rats (postnatal 1–2 days) were digested with HBSS containing 0.1% trypsin (Invitrogen, Carlsbad, CA, United States) and 0.05% type-II collagenase (Worthington, Lakewood, CO, United States). Cell suspensions were then collected and pre-plated for 2 h to remove fibroblasts, and then were rinsed twice with culture medium and 0.1 μM 5-bromo-2′-deoxyuridine (Brdu) was added, the supernatant containing purified cardiomyocytes was collected and cultured for another 48 h before treating. Immunofluorescence analysis was performed by using the cardiomyocyte specific marker α-actinin to ensure the purity of cardiomyocytes without cardiac fibroblasts nor endothelial cells.

### Oxygen-glucose deprivation model

The oxygen-glucose deprivation (OGD) is commonly used as the cell model of hemorrhagic shock (Shu et al., 2022). Briefly, isolated rat neonatal cardiomyocytes were cultured in DMEM with 10% FBS at 37°C for 48 h and reaching 80% confluency before treated. Then the culture medium was changed to glucose-free DMEM (Invitrogen) without FBS. At the same time, cells were placed in a hypoxia incubator chamber (Coolrun, China) containing 95% N_2_ and 5% CO_2_ at 37°C. The chamber was sealed and placed in an incubator for additional 6 h of OGD. For the normal glucose oxygen group, the high-glucose medium containing 10% FBS was used in the ordinary chamber as control.

### Transmission electron microscopic analysis

Fresh rat heart tissue samples were fixed overnight at 4°C with 2% glutaraldehyde natrium cacodylicum solution (0.1 M, pH 7.2). The samples were then fixed in 1% osmium tetroxide aqueous solution for 1 h. The digital images were acquired using the JEM-1230 high-contrast transmission electron microscope and soft imaging system (JEOL, Tokyo, Japan). ImageJ software (US National Institutes of Health) was used to quantify the ultrastructure of mitochondria. Mitochondria volume density was determined with a transparent grid overlaying electron micrograph and the volume density of mitochondria = the number of grid points falling into mitochondria/the number of total points (Medeiros, 2008). Mitochondria cristae score was performed [0—no sharply defined cristae; 1—greater than 50% of the mitochondrial area without cristae; 2—greater than 25% of mitochondrial area without cristae; 3—many cristae (over 75% of area) but with irregular arrangement; 4—many regular cristae] (Eisner et al., 2017; Lam et al., 2021). At least 200 mitochondria from 5 rats were analysed in each group.

### Measurement of mitochondrial DNA copy number

Whole blood of the rats was taken 12 h after HS. Genomic and mitochondrial DNA was extracted from blood by using the tissue genomic DNA miniprep kit (Beijing Zoman Biotech, China). mtDNA was quantified by relative copy number ratio of mtDNA to genomic DNA. The cytochrome B (Cyt B) gene was used for determining the mtDNA level as previously described (Zhang et al., 2010), and Gapdh gene for the genomic DNA. RT-PCR was performed using SYBR green fluorescence (Thermo Fisher, United States). The DNA levels were determined with the following primers: Cyt B specific primers (5′-TCC​ACT​TCA​TCC​TCC​CAT​TC-3′ and 5′-CTG​CGT​CGG​AGT​TTA​ATC​CT-3′); Gapdh specific primers (5′-GAA​ATC​CCC​TGG​AGC​TCT​GT-3′ and 5′-CTG​GCA​CCA​GAT​GAA​ATG​TG-3′) (Zhang et al., 2010). All data were quantified by use of the comparative CT method.

### Reactive oxygen species content detection

For rat myocardial tissue, the fresh heart tissues were collected 12 h after HS. The high-quality living-tissue oxidative stress ROS fluorescence assay kit (HalingBio Co., Ltd., Shanghai, China) was used by to detect the ROS content. Primary rat neonatal cardiomyocytes were cultured in confocal dishes, and were incubated with 5 μM mitoSOX Red (Invitrogen) or 20 μM DCFH-DA (Sigma, St. Louis, Mo, United States) at 37°C for 30 min and washed twice in PBS. The images were observed and photographed using an immunofluorescence microscope (TCS-SP8; Leica, Wetzlar, Germany). The fluorescence intensity was determined using ImageJ.

### Library construction and mRNA sequencing

After extracting mRNA by using the TRIzol^®^ Reagent (Invitrogen) and inspecting the isolated mRNA, genomic DNA was removed using DNase I (TaKara). The TruSeq™ RNA sample preparation Kit from Illumina (San Diego, CA) was used to generate the sequencing library. The index coding samples were clustered, and the library was prepared and sequenced with the Illumina HiSeq xten/NovaSeq 6,000 sequencer (2 × 150 bp read length). The raw data of RNA-seq were uploaded to Sequence Read Archive (SRA) database and the BioProject ID is: PRJNA880976.

### Differential expression analysis

For mRNA, we downloaded the UCSC (RAT RGSC 6.0/rn6) reference genome and gene model annotation files. HISAT2 (version 2.0.4) was used for comparison with the reference genome. String Tie (version 2.1) was used to calculate the fragments per kilobase per million (FPKM) of mRNA transcription. Digital Gene Expression in R, (http://www.bioconductor.org/packages/2.12/bioc/html/edgeR.html), was utilized for differential expression analysis. The *p*-value was adjusted using the Q value. For significant differential expression, *p* < 0.05 and |log_2_FC|≥1were used as thresholds to screen more DEGs, and the Q value was used as a reference for further studying the target genes.

### Comparison with the rat gene database

We searched and downloaded the rat inflammatory response gene data set GO: 0006954, proinflammatory gene data set GO: 0050729, and anti-inflammatory gene data set GO: 0050728 from the rat gene database RGD (https://rgd.mcw.edu/). These datasets were compared with the DEGs. The screening criteria for DEGs were: *p* < 0.05 and |log_2_FC|≥1.

### Statistical analysis

All data are expressed as mean ± SEM except for RNA-seq data. Statistical significance of differences between groups was calculated by unpaired *t*-test or one-way ANOVA followed by Student-Newman-Keuls analysis of variance. *p* < 0.05 was considered statistically significant.

## Results

### Hemorrhagic shock damages the myocardial mitochondrial ultrastructure and increases the mitochondrial DNA release

**FIGURE 1:**
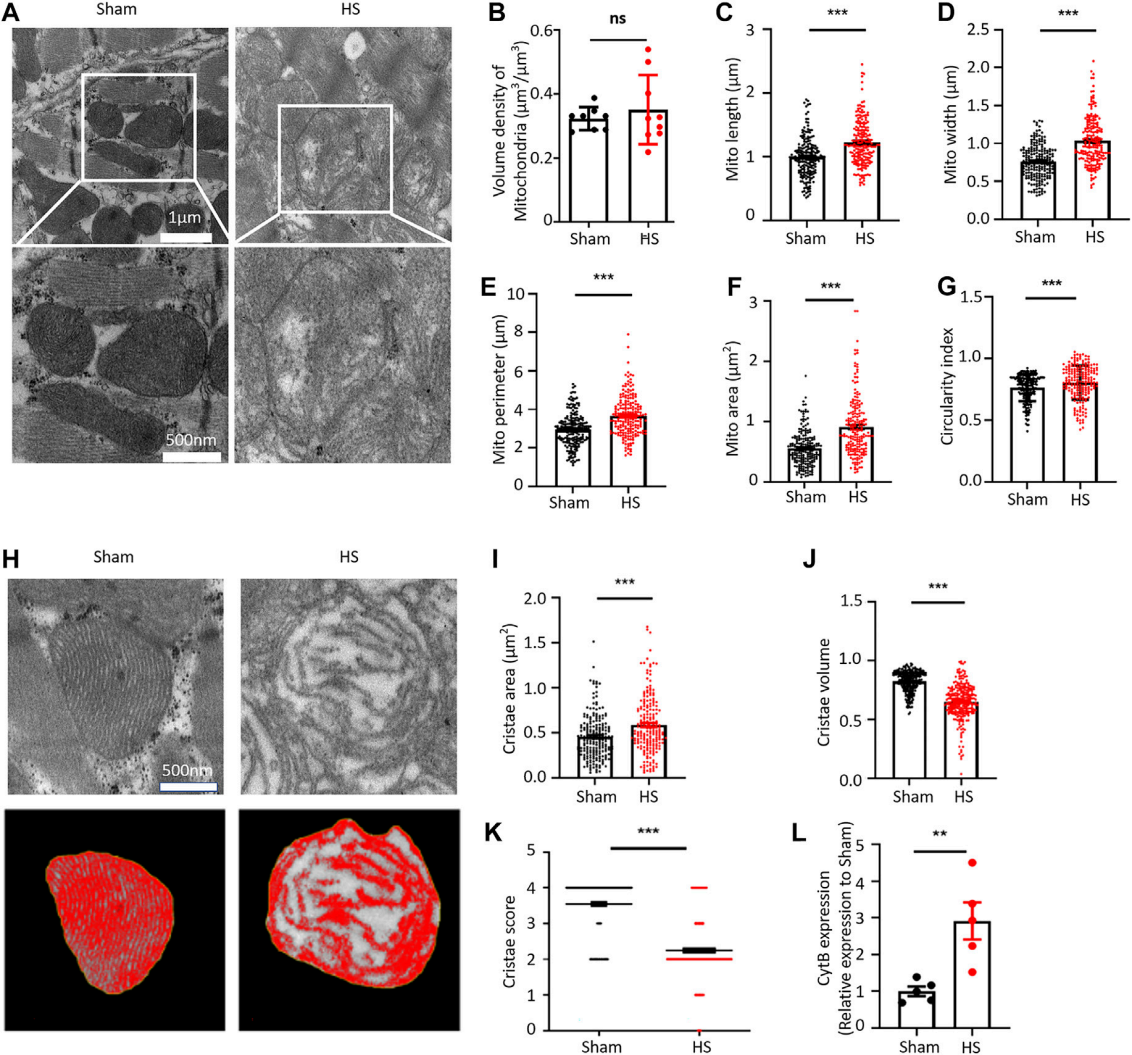
HS damages the myocardial mitochondrial ultrastructure and increases the mtDNA release. **(A)** Representative transmission electron microscopic (TEM) images of mitochondria in the myocardial tissue of rats from the sham group and HS group. **(B**–**G)** Statistical results of the myocardial mitochondrial volume density, length, width, perimeter, area, and circularity index in the sham group and HS group. ImageJ was used to quantitatively analyse the myocardial mitochondrial morphology, *n* = 200 mitochondria from 5 rats in each group, ***p < 0.001 compared with the sham group. **(H)** Representative TEM images of myocardial mitochondrial cristae ultrastructure of the sham group and HS group rats. **(I**–**K)** Statistical results of the mitochondrial cristae area, cristae volume, and cristae score, *n* = 200 mitochondria from 5 rats per group, ***p < 0.001 compared with the sham group. **(L)** Statistical results of the mitochondrial DNA CytB content in peripheral blood of the sham group and HS group. *n* = 5 rats per group, ***p < 0.01 compared with the sham group.

We first established a 40% fixed-blood-loss HS rat model (Supplementary Figures 1, 2; Supplementary Tables 1, 2) and then observed the ultrastructure of myocardial tissue through transmission electron microscope (TEM). Interestingly, at 12 h after HS, cardiac mitochondrial ultrastructure was found to be swollen and disrupted, although the arrangement of myofilaments was still regular (Figure 1A). By using the “point counting grids” methods (Supplementary Figure 3), we found no significant change of the mitochondrial volume density in the myocardium 12 h after HS (Figure 1B); However, specifically, we observed that the average mitochondrial length, width, perimeter, and mitochondrial area were significantly increased in the HS group compared with those in the sham group (Figures 1C–F). Consistently, the average circularity index of mitochondria in HS myocardium increased to 0.804 compared with that of 0.762 in sham (Figure 1G), indicating that mitochondria had swollen and became rounder in the myocardium 12 h after HS. We further assessed the cristae morphology of mitochondria in TEM images and found that the cristae were disrupted, which was manifested by the reduced cristae volume density and more irregular morphology (Figure 1H). Although the average cristae surface area was increased to 0.585 ± 0.023 µm^2^ after HS compared with that of 0.456 ± 0.017 µm^2^ in the sham group due to mitochondrial swelling (Figure 1I), the mitochondrial cristae volume density (refers to the mitochondrial cristae area/mitochondrial area ratio) significantly decreased to 0.642 ± 0.011 after HS compared with that of 0.824 ± 0.006 in the sham group (Figure 1J). Furthermore, the cristae score was markedly reduced after HS relative to the sham group (Figure 1K). These results indicated that the ultrastructure of myocardial mitochondria is damaged after HS. A previous study clearly demonstrated that dysfunctional mitochondria are the crucial sources of DAMPs (Jansen et al., 2018). Mitochondrial damage and dysfunction lead to the destruction and release of mtDNA. Therefore, we determined the mtDNA content in blood of rats after HS and found that the content of the mtDNA marker Cytochrome B (CytB) in blood of the HS rats was significantly increased (Figure 1L). These results demonstrated that myocardial mitochondrial ultrastructure was damaged and the mtDNA release was increased after HS.

### Differentially expressed genes of myocardial tissues after hemorrhagic shock are significantly enriched in mitochondrial and reactive oxygen species pathways

**FIGURE 2:**
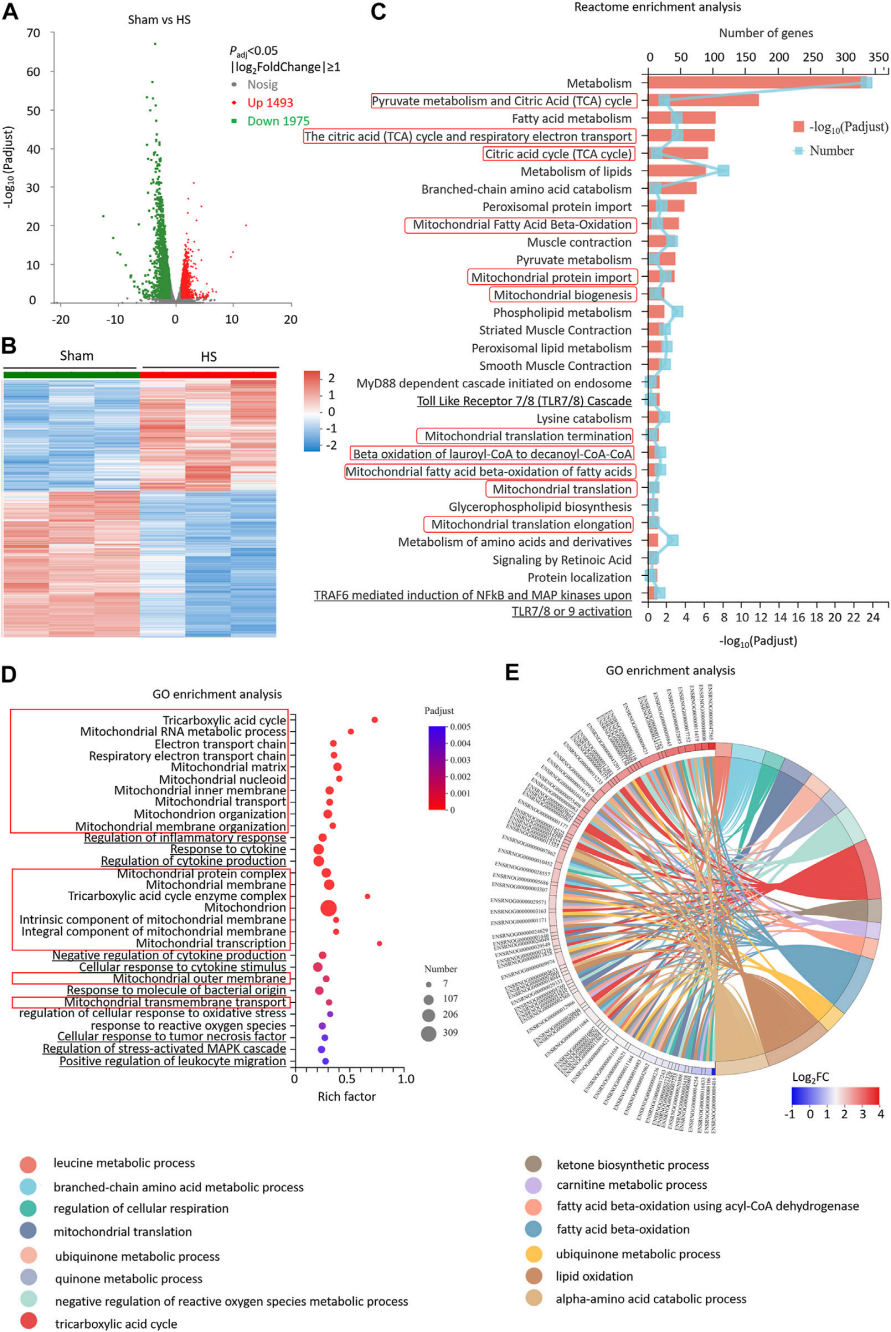
Differentially expressed genes of myocardial tissues after HS are significantly enriched in mitochondrial and ROS pathways. **(A)** The volcano plot showing number of differentially expressed genes (DEGs) of HS and sham rat heart tissues; *P*_adj_ < 0.05, *n* = 3 rats per group. **(B)** A heat map summary of the DEGs expression values of HS and sham group. Red indicates high and green indicates low gene expression *P*_adj_ < 0.05, *n* = 3 mice per group. **(C)** Reactome enrichment analysis and **(D)** GO enrichment analysis of all the DEGs and GO cluster plot showing a chord dendrogram **(E)** of the clustering of the expression spectrum of the 309 DEGs in the mitochondrion GO term between sham and HS.

We further investigated the whole transcriptome of HS and sham mouse heart tissues by using Illumina RNA-Seq technology to screen the differentially expressed genes (DEGs) and potential signaling pathways after HS. The cardiac tissues were taken 12 h after HS for mRNA high-throughput sequencing, and a total of 16,831 mRNAs were detected. Compared with the sham mouse hearts, 3,468 DEGs were identified in the HS group, of which 1,493 were upregulated and 1975 were downregulated (*p* < 0.05, |log_2_FC| ≥ 1, *n* = 3, Figures 2A,B). Moreover, the Reactome enrichment analysis of the DEGs showed that metabolic pathways, especially TCA cycle, mitochondrial fatty acid beta-oxidation, mitochondrial protein import, mitochondrial biogenesis and mitochondrial translation (marked with red boxes) were involved in most of the top significant enrichment pathways (*P*_adj_ < 0.05, Figure 2C). We further enriched DEGs according to their function through GO enrichment analysis and found the total number of enriched GO terms with significant differences (*p* < 0.05) was 1,127, in order to avoid miscellaneous information interfering with the topic of this study, we picked up 30 GO terms of interest mainly involving mitochondrion-related (marked with red boxes), inflammation-related (marked with underline) and ROS-related pathways. In HS rat hearts, Tricarboxylic acid cycle, Mitochondrion, electron transport chain, mitochondrial RNA metabolic process, et al. were all involved in most of the top significant enrichment function terms (*P*_adj_ < 0.05, Figure 2D). In addition, ROS-related pathways including regulation of cellular response to oxidative stress and response to reactive oxygen species were also significantly enriched. Since 309 DEGs were significantly enriched in the mitochondrion GO term (GO: 0005739), we further plotted a chord dendrogram of the clustering of the expression spectrum of the 309 DEGs enriched in the mitochondrion GO term between the sham and HS groups (*P*_adj_ < 0.05, Figure 2E). Collectively, the aforementioned data demonstrated that the DEGs of myocardial tissues after HS were significantly enriched in mitochondrial and ROS-related pathways.

### SkQ1 attenuates the increased reactive oxygen species induced by hemorrhagic shock in myocardial tissues and by oxygen-glucose deprivation in cardiomyocytes

**FIGURE 3:**
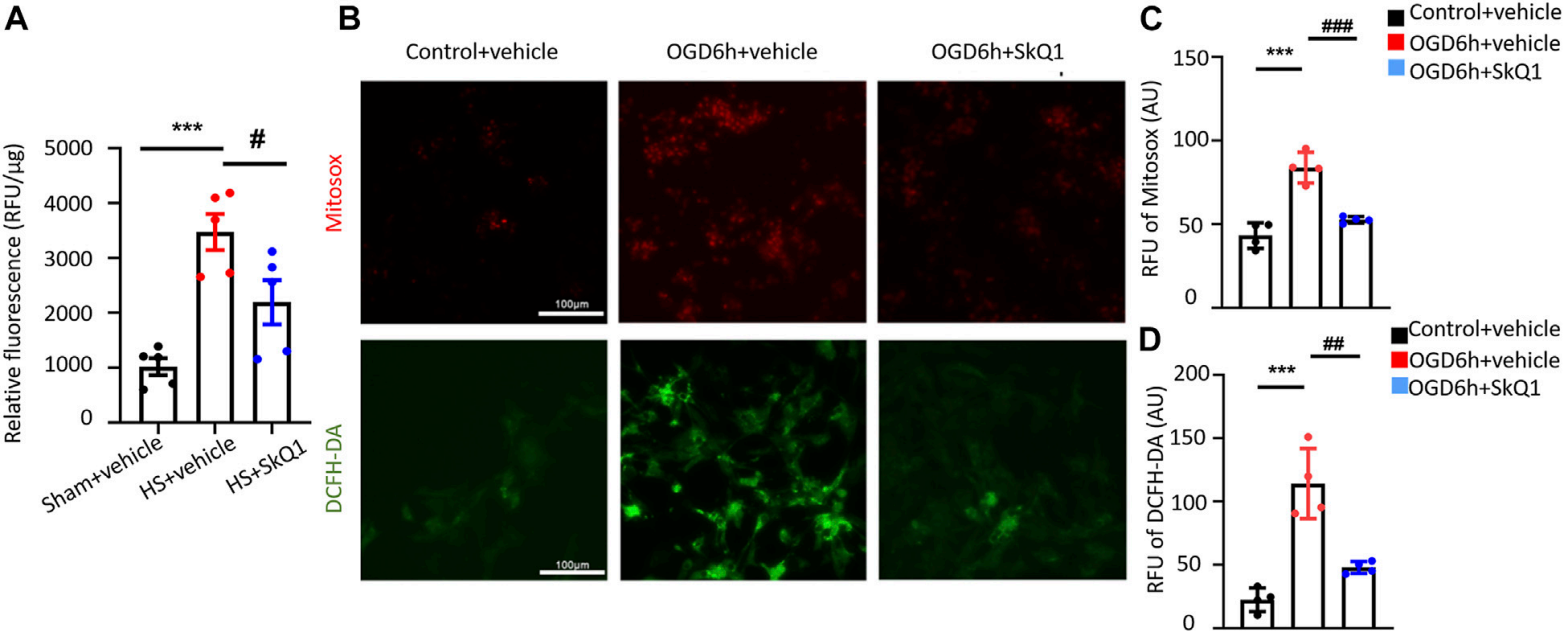
SkQ1 attenuates the increased ROS induced by HS in myocardial tissues and by oxygen-glucose deprivation in cardiomyocytes. **(A)** Statistical relative fluorescence units (RFU) of the myocardial ROS levels in the sham, HS + vehicle and HS + SkQ1 groups. *n* = 5 mice per group; ^***^ *p* < 0.001 vs. the sham; and ^#^ *p* < 0.05 vs. HS + vehicle. **(B)** Representative images of ROS fluorescence staining of neonatal rat cardiomyocytes from the control + vehicle, OGD 6 h + vehicle, and OGD 6 h + SkQ1 group. MitoSOX was used to label mitochondrial ROS (red fluorescence), and DCFH-DA was used to label cytoplasmic ROS (green fluorescence). **(C,D)** Statistics MitoSOX and DCFH-DA RFU of the ROS fluorescence intensity in rat cardiomyocytes. *n* = 3 independent experiments, ^***^ *p* < 0.001 vs. the control + vehicle; and ^##^ *p* < 0.01, ^###^ *p* < 0.001 vs. OGD 6 h + vehicle.

The aforementioned results showed that HS causes damage to the myocardial mitochondrial ultrastructure, increases mtDNA release, and results in significant enrichment of differentially expressed genes in the mitochondrial and ROS-related pathways. However, whether mitochondria-targeted protective drugs especially antioxidant can ameliorate the damage to the heart after HS remains unclear. As the first mitochondria-targeted antioxidant drug in clinical application (Yani et al., 2012), SkQ1 (Visomitin) has been reported to effectively eliminate excessive intracellular ROS and exert anti-inflammatory effects (Plotnikov et al., 2010; Kapay et al., 2011; Isaev et al., 2012; Kirpatovsky et al., 2013; Genrikhs et al., 2015). Nevertheless, the specific role of SkQ1 in HS has not yet been explicated. Therefore, we treated HS rats with SkQ1 and determined the effect of SkQ1 on myocardial ROS after HS. After establishing the rat HS model, the rats in the HS + SkQ1 group were intraperitoneally injected with SkQ1 (1 μM/kg) immediately after HS surgery, whereas those in the HS + vehicle group were injected with the same dose of vehicle. The content of myocardial ROS was detected 12 h after HS. Obviously, SkQ1 effectively attenuated the HS-induced increase in the myocardial ROS content (Figure 3A), suggesting an inhibitory effect of SkQ1 on myocardial ROS after HS. In addition, we isolated the neonatal rat cardiomyocytes and simulated cells with the oxygen-glucose deprivation (OGD) for 6 h as the cell model commonly used of hemorrhagic shock. MitoSOX and DCF were used to detect mitochondrial and cytosolic ROS respectively. Consistently, SkQ1 effectively attenuated the OGD-induced increase in mitochondrial and cytosolic ROS in cardiomyocytes (Figures 3B–D). The aforementioned results demonstrated that SkQ1 attenuates the increased ROS induced by HS in myocardial tissues and by OGD in cardiomyocytes consistent with its anti-oxidation effects (Zinovkin and ZAMYATNIN, 2019).

### SkQ1 protects the ultrastructure of myocardial mitochondria and attenuates mitochondrial DNA release after hemorrhagic shock

**FIGURE 4:**
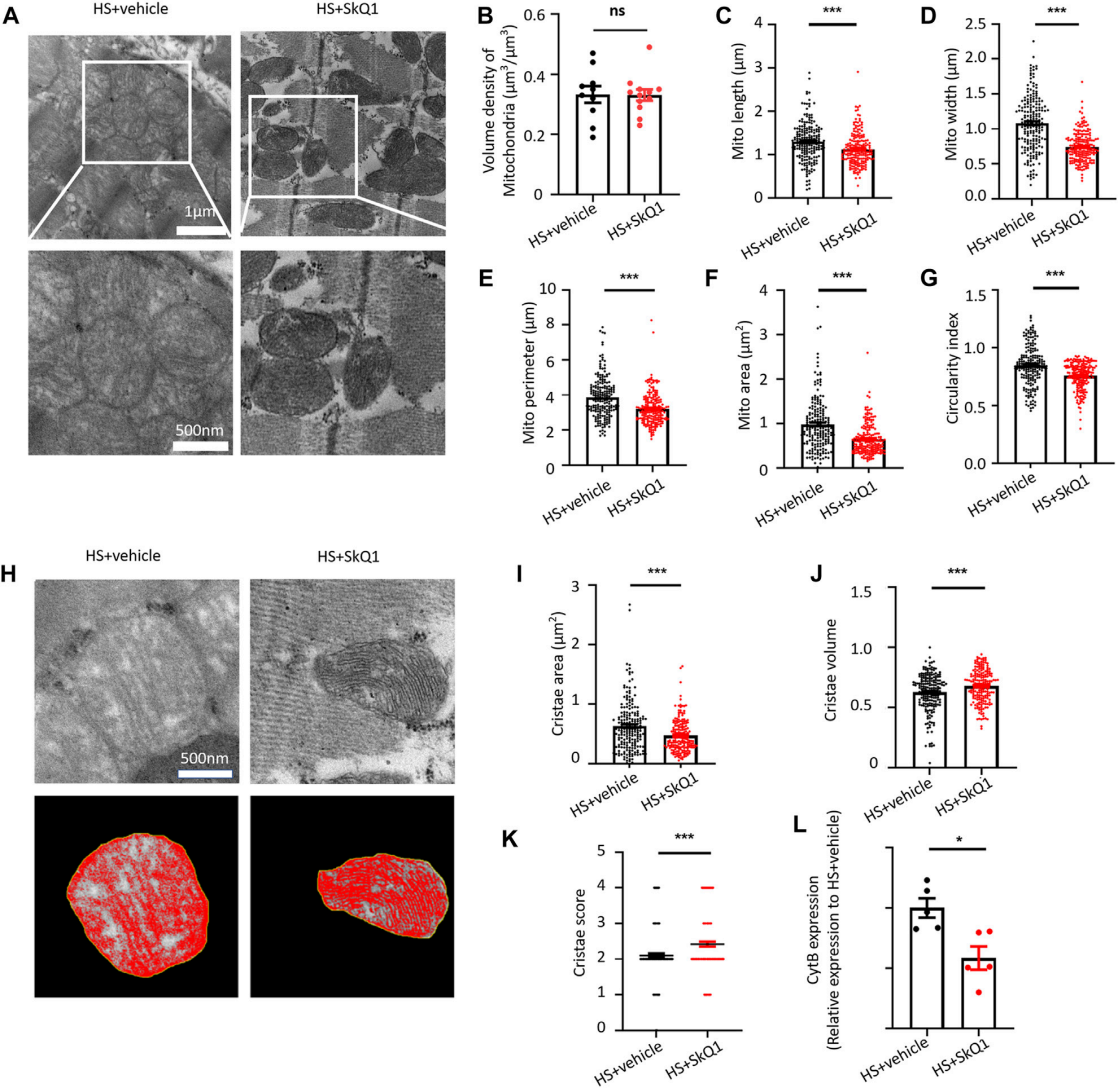
SkQ1 protects the ultrastructure of myocardial mitochondria and attenuates mtDNA release after HS. **(A)** Representative TEM images of mitochondria of the rat myocardial tissues from the HS + vehicle and HS + SkQ1 group rats. **(B**–**G)** Statistical results of the myocardial mitochondrial volume density, length, width, perimeter, area, and circularity index in the HS + vehicle and HS + SkQ1 group, *n* = 200 mitochondria from 5 rats per group, ***p < 0.001 compared with the HS + vehicle group. **(H)** Representative TEM images of myocardial mitochondrial cristae ultrastructure of the HS + vehicle and HS + SkQ1 group. **(I**–**K)** Statistical results of the mitochondrial cristae area, cristae volume, and cristae score, *n* = 188–200 mitochondria from 5 rats per group, ***p < 0.001 compared with the HS + vehicle group. **(L)** Statistical results of the mitochondrial DNA CytB content in peripheral blood of the HS + vehicle and HS + SkQ1 group. *n* = 5 rats per group, *p < 0.05 compared with the sham group.

We further observed the effect of SkQ1 on the ultrastructure of myocardial tissue through transmission electron microscope (TEM) and found that the swelling degree of myocardial mitochondria in HS was alleviated after SkQ1 treatment (Figure 4A). Specifically, a slight change was observed in mitochondrial volume density of the myocardium 12 h after SkQ1 treatment (Figure 4B). However, the increased mitochondrial length, width, perimeter, and mitochondrial area after HS were all significantly reduced in the HS + SkQ1 group compared with those in the HS + vehicle group (Figures 4C–F). The average circularity index of mitochondria in HS + SkQ1 group myocardium decreased to 0.755 compared with that of 0.846 in the HS + vehicle group (Figure 4G), indicating that SkQ1 ameliorates mitochondrial swelling and rounding in the myocardium 12 h after HS. We further assessed the cristae morphology of mitochondria in TEM images and found that SkQ1 protects against the disruption of cristae morphology after HS (Figure 4H). Although the average cristae surface area was decreased to 0.468 ± 0.020 µm^2^ in the HS + SkQ1 group compared with that of 0.628 ± 0.030 µm^2^ in the HS + vehicle (Figure 4I), the mitochondrial cristae volume density significantly increased to 0.677 ± 0.010 in the HS + SkQ1 compared with that of 0.624 ± 0.011 in the HS + vehicle group (Figure 4J). Furthermore, the cristae score was markedly increased after SkQ1 treatment relative to the HS + vehicle group (Figure 4K). These results indicated that SkQ1 alleviates damage to the ultrastructure of myocardial mitochondria after HS. Furthermore, we determined the mtDNA content in blood of rats and found that the content of the mtDNA marker CytB in blood of the HS rats was significantly decreased (Figure 4L). These results demonstrated that myocardial mitochondrial ultrastructure was damaged and the mtDNA release was increased after HS.

### SkQ1 treatment after hemorrhagic shock causes DEGs of myocardial tissues enriched in the mitochondrial and reactive oxygen species related pathways

**FIGURE 5:**
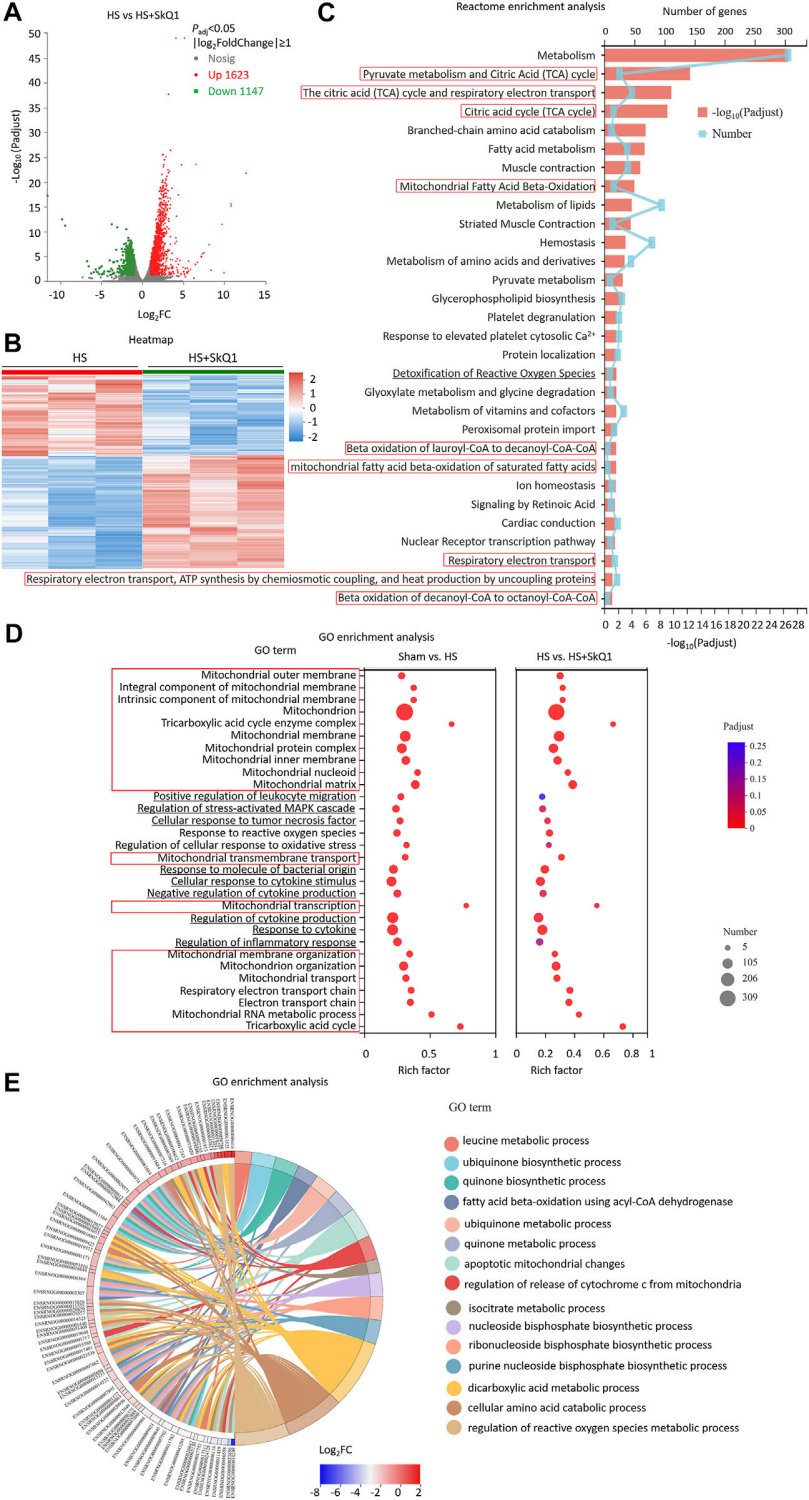
SkQ1 treatment after HS causes DEGs of myocardial tissues enriched in the mitochondrial and ROS-related pathways. **(A)** The volcano plot showing number of differentially expressed genes (DEGs) of HS + SkQ1 and HS rat heart tissues; *P*_adj_ < 0.05, *n* = 3 rats per group. **(B)** A heat map summary of the DEGs expression values of HS + SkQ1 and HS group. Red indicates high and green indicates low gene expression *P*_adj_ < 0.05, *n* = 3 mice per group. **(C)** Reactome enrichment analysis and **(D)** GO enrichment analysis of all the DEGs and GO cluster plot showing a chord dendrogram **(E)** of the clustering of the expression spectrum of the 278 DEGs in the mitochondrion GO term between HS + SkQ1 and HS.

We further screened the DEGs between the HS + SkQ1 and HS groups. A total of 2,770 DEGs were screened, of which 1,623 exhibited an increased expression and 1,147 exhibited a decreased expression (*P*_adj_ < 0.05, |log_2_FC| ≥ 1, Figures 5A,B). Moreover, the Reactome enrichment analysis of the DEGs showed that metabolism especially TCA cycle, mitochondrial fatty acid beta-oxidation, respiratory electron transport, ATP synthesis by chemiosmotic coupling, and heart production by uncoupling (marked with red boxes) were involved in most of the top significant enrichment pathways (*P*_adj_ < 0.05, Figure 5C). We further enriched DEGs according to their function through GO enrichment analysis and found that mitochondrial pathways including mitochondrial outer membrane, mitochondrion, mitochondrial membrane organization, mitochondrial RNA metabolic process, TCA cycle, et al. (marked with red boxes), and the ROS pathways including response to reactive oxygen species which were enriched between HS and sham were still involved in significant enrichment GO terms between the HS + SkQ1 and HS groups (*P*_adj_ < 0.05, Figure 5D), except 4 pathways were no longer significantly enriched including regulation of stress-activated MAPK cascade (*P*_adj_ = 0.057), regulation of cellular response to oxidative stress (*P*_adj_ = 0.084), regulation of inflammatory response (*P*_adj_ = 0.122), and positive regulation of leukocyte migration (*P*_adj_ = 0.258) (Figure 5D). Since 278 DEGs were significantly enriched in the mitochondrion terms, we further plotted a chord dendrogram of the clustering of the expression spectrum of the 278 DEGs enriched in the mitochondrion GO term between the HS and HS + SkQ1 groups (*P*_adj_ < 0.05, Figure 5E). Collectively, the aforementioned data demonstrated that SkQ1 treatment after HS causes enrichment of DEGs of myocardial tissues in the mitochondrial and ROS-related pathways.

### SkQ1 improves inflammatory response after hemorrhagic shock

**FIGURE 6:**
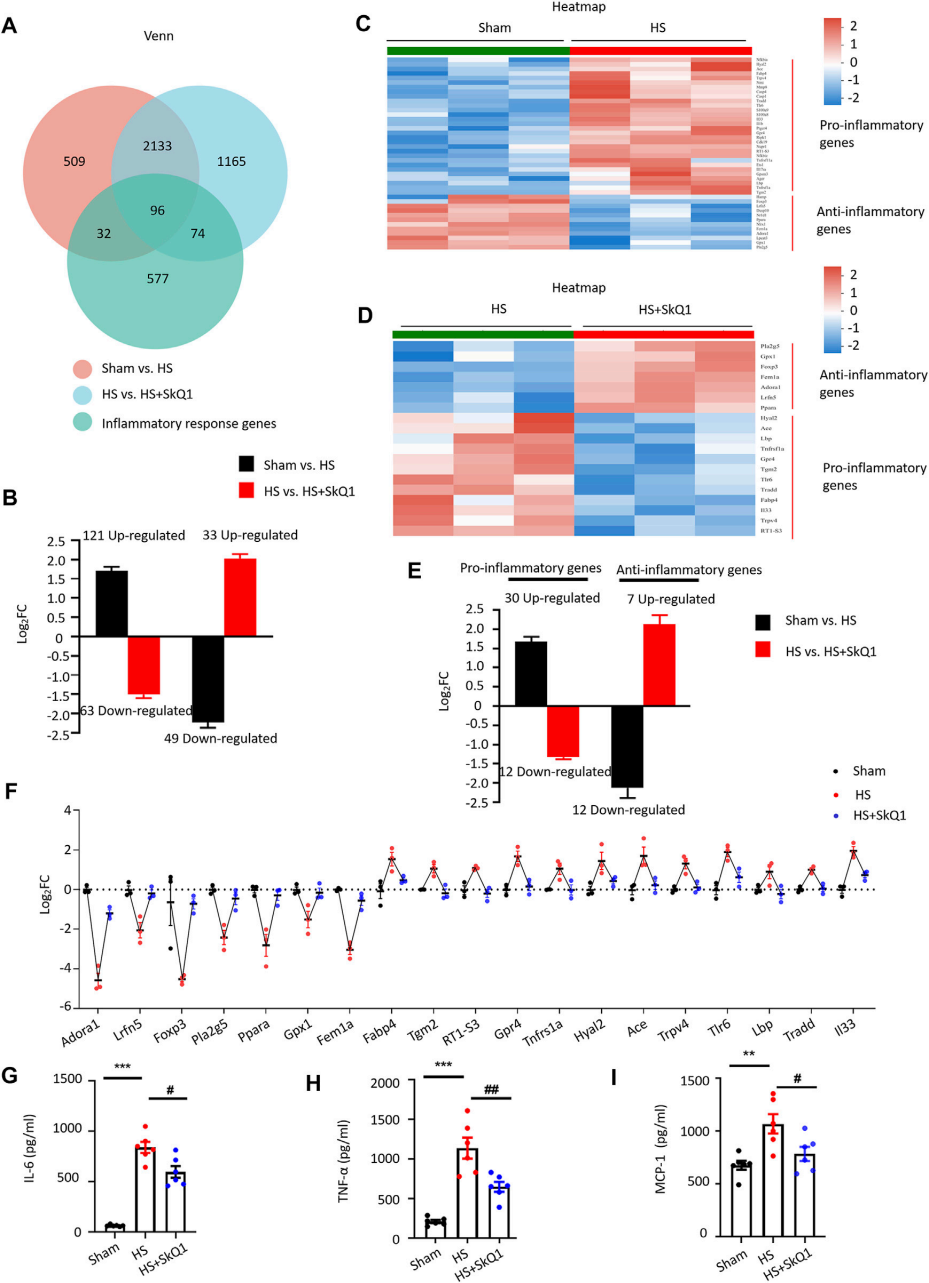
SkQ1 improves inflammatory response after HS. **(A)** Venn diagram of the inflammatory response DEGs in the sham vs. HS and in the HS + vehicle vs. HS + SkQ1 group; the expressions of 96 inflammatory genes were reversed by SkQ1. **(B)** Statistical results of the average expression of inflammatory response genes. The black column chart shows the changes in inflammatory gene expression in the HS vs. sham group. The red column chart shows the changes in inflammatory gene expression in the HS + SkQ1 vs. HS + vehicle group; *n* = 3 mice per group; log_2_FC > 0 indicates gene expression increased, and log_2_FC < 0 indicates gene expression decreased. **(C)** Hierarchical cluster analysis heatmap of myocardial proinflammatory and anti-inflammatory response DEGs in the sham and HS group. **(D)** Hierarchical cluster analysis heatmap of the myocardial proinflammatory and anti-inflammatory response DEGs in the HS + vehicle and HS + SkQ1 group. **(E)** Average expression of proinflammatory and anti-inflammatory response genes: The black column chart shows the changes in inflammatory gene expression in the HS vs. sham group. The red column chart shows the changes in inflammatory gene expression in the HS + SkQ1 vs. HS + vehicle group; **(F)** Statistical results of the 7 anti-inflammatory and 12 pro-inflammatory genes change in the sham, HS, and HS + SkQ1 groups; *n* = 3 mice per group; log_2_FC > 0 indicates gene expression increased, and log_2_FC < 0 indicates gene expression decreased. **(G**–**I)** Statistical results of the protein expression of proinflammatory factors, TNF-α, IL-6, and MCP-1 in blood determined through ELISA; *n* = 6 mice per group; ^**^ *p* < 0.01, ^***^ *p* < 0.001 vs. sham + vehicle group; ^#^ *p* < 0.05, ^##^ *p* < 0.01 vs. HS + vehicle group.

According to GO enrichment analysis, in addition to the mitochondrial signals, the inflammatory response signals such as positive regulation of leukocyte migration, regulation of stress-activated MAPK cascade, cellular response to tumor necrosis factor and cytokine stimulus, et al. (marked with black underlines) were significantly enriched after HS treatments (Figure 2D) and response to cytokine, cellular response to tumor necrosis factor and negative regulation of cytokine production, et al. were significantly enriched after SkQ1 treatments compared with HS group (Figure 5D). We overlapped the DEGs with rat inflammatory response gene data set (GO: 0006954) from the rat gene database RGD (https://rgd.mcw.edu/) and obtained 128 overlapping genes, of which 41 exhibited 4.16 ± 0.35-fold (range = 2.01–10.68 folds) increased expression and 87 exhibited 3.40 ± 0.48-fold (range = 2.02–37.69 folds) decreased expression (Figure 6A). Importantly, the expression of 63 of the 121 genes, which exhibited an increased expression in the HS vs. sham group, was decreased in the HS + SkQ1 vs. HS group by 3.60 ± 0.64 folds (range = 2.03–39.71 folds). Moreover, 33 of the 49 genes that exhibited a decreased expression in the HS group showed 4.54 ± 0.40-fold (range = 2.06–10.68 folds) increased expression in the HS + SkQ1 group (Figure 6B). In short, compared with the sham group, the expression of 96 (56.5%) of the 170 genes with an altered transcription level in the HS group could be significantly restored in the HS + SkQ1 group, suggesting that SkQ1 improves the inflammatory response to heart damage after HS.

Moreover, by comparing the expression of DEGs with that of the pro-inflammation- and anti-inflammation-related gene set from RGD (GO: 0050729 and GO: 0050728), we found that in the sham vs. HS group, the expression of 12 anti-inflammatory genes decreased by 5.43 ± 1.38 fold (range = 2.02–19.45 folds), and the expression of 30 proinflammatory genes increased by 3.67 ± 0.47 fold (range = 2.02–13.40 folds). Seven (58.3%) of the 12 anti-inflammatory genes whose expression was decreased in the HS vs. sham group exhibited a 4.76 ± 0.86-fold (range = 2.35–8.86 folds) increased expression in the HS + SkQ1 group. Additionally, 12 (40%) of the 30 pro-inflammatory genes whose expression was increased in the HS vs. sham group exhibited a 2.53 ± 0.10-fold (range = 2.10–3.35 folds) decrease in expression in the HS + SkQ1 group (Figures 6C–E). We further used scatter plot to show the individual change of 7 anti-inflammatory genes and 12 pro-inflammatory genes of the sham, HS, and HS + SkQ1 groups (Figure 6F). Obviously, the 7 decreased anti-inflammatory genes including Adenosine Receptor A1 (Adora1), Leucine Rich Repeat And Fibronectin Type III Domain Containing 5 (Lrfn5), Forkhead Box P3 (Foxp3), Phospholipase A2 Group V (pla2g5), Peroxisome Proliferator Activated Receptor Alpha (Ppara), Glutathione Peroxidase 1 (Gpx1), Fem-1 Homolog A (Fem1a) in the HS vs. sham group were increased after SkQ1 treatment. The increased 12 pro-inflammatory genes including Fatty Acid Binding Protein 4 (Fabp4), Transglutaminase 2 (Tgm2), RT1 class Ib locus S3 (RT1-S3), G protein-coupled receptor 4 (Gpr4), TNF Receptor Superfamily Member 1A (Tnfrsf1a), Hyaluronidase 2 (Hyal2), Angiotensin I Converting Enzyme (Ace), Transient Receptor Potential Cation Channel Subfamily V Member 4 (Trpv4), Toll Like Receptor 6 (Tlr6), Lipopolysaccharide Binding Protein (Lbp), Tumor Necrosis Factor Receptor Type 1-Associated DEATH Domain Protein (Tradd), Interleukin 33 (Il33) in the HS vs. sham group were decreased after SkQ1 treatment.

To further determine the expression of inflammatory factors in blood at the protein levels, rat peripheral blood samples were collected and the protein levels of TNF-α, IL-6, and MCP-1 were detected by ELISA. We found that 12 h after HS, the TNF-α, IL-6, and MCP-1 levels were significantly increased systematically compared with those in sham. SkQ1 significantly reversed the HS-induced increases in the Tumor necrosis factor-alpha (TNF-α), Interleukin-6 (IL-6), and Monocyte chemotactic protein 1 (MCP-1) (Figures 6G–I), suggesting that SkQ1 effectively improves the inflammatory response after HS.

## Discussion

Cell hypoxia and local inflammation lead to increased ROS production in cell mitochondria, cause oxidative damage related to the mitochondrial respiratory chain and mitochondrial dysfunction, and induce the release of mtDNA fragments in many diseases including HS (Aswani et al., 2018; Zhao et al., 2021a). Although mitochondrial damage and the release of mtDNA fragments occur in several trauma disease models (Zeng et al., 2016; Aswani et al., 2018; Carter et al., 2019; Zhao et al., 2021b), the effects of mitochondria in cardiomyocytes after HS are unclear yet. In the present study, we found that HS caused damage to the rat myocardial mitochondrial structure, increased mtDNA and ROS contents, and led to the activation of the mitochondrial pathways and systemic inflammatory response. Moreover, we uncovered a novel protective role of the mitochondrial antioxidant SkQ1, that is, decreasing inflammation by protecting myocardial mitochondria after HS.

Changes in the mitochondrial ultrastructure mainly involve alterations in the mitochondrial morphology (Li et al., 2015) [manifested by an increase in the mitochondrial area, a decrease in the length/width ratio, and an increase in circularity index (Zhao et al., 2021b)] and destruction of the mitochondrial cristae structure (Lam et al., 2021) (manifested by a decrease in the cristae volume, represented by mitochondrial cristae area/mitochondrial area). The present study is the first to report the changes of mitochondrial morphology and mitochondrial cristae structure in cardiomyocytes of the HS rats, which supports the conclusion that HS leads to myocardial mitochondrial damage. Our study also showed that the mtDNA content in blood of the rats with severe HS (blood loss >40% of the total blood volume) increased significantly 12 h after HS, which is consistent with the reported increase in the mtDNA content in blood of the rats with traumatic HS. Yet, previous studies have shown that in animals with simple HS, that is, mild-to-moderate blood loss (<30% of total blood volume), the mtDNA content is not significantly increased (Aswani et al., 2018), which may be related to the fact that under mild-to-moderate HS, the body can ensure tissue perfusion through the compensatory mechanism.

The antioxidant SkQ1 is the first mitochondrial-targeted drug to be used in medical practice (Yani et al., 2012). Studies have reported that it can effectively remove excessive ROS in various models and alleviate the adverse effects (Zhao et al., 2005; Skulachev, 2007; Isaev et al., 2012; Yani et al., 2012; Genrikhs et al., 2015). However, whether SkQ1 can play a positive role in HS treatment remains to be investigated. We found that SkQ1 attenuated the increased ROS production induced by HS in myocardial tissues and by OGD in cardiomyocytes. Ultrastructurally, SkQ1 protected the myocardial mitochondrial structure and reduced the release of the peripheral blood mtDNA after HS. RNA-seq transcriptome analysis showed that the expression of 56.5% of the inflammation-related genes, which altered after HS, could be significantly reversed after SkQ1 treatment. Moreover, SkQ1 significantly reversed the HS-induced increases in the TNF-α, IL-6, and MCP-1 levels in rat peripheral blood detected through ELISA. The effect of SkQ1 on mitochondria is systemic, and mtDNA release is also a result of mitochondrial damage and inflammation induction in various tissues. The limitation of the present study is that it only determined the protective effect of SkQ1 on the myocardium. Nevertheless, the protective effect on the myocardial mitochondria only partially explains the reason for the decrease in mtDNA release. The contribution of mitochondrial damage in other tissues to the blood mtDNA release after HS remains to be further studied.

Some studies have shown that MTDs, including mtDNA, is released in large quantities after mitochondrial damage and is recognised by toll-like receptors, leading to NF-κB activation (Zhang et al., 2018) and further induces transcriptional activation of inflammatory cytokines and NLRP3 and initiates aseptic inflammation (Zhong et al., 2018; De Gaetano et al., 2021). By analyzing the changes in the transcriptional level of inflammatory genes in heart tissues after HS through high-throughput sequencing of heart tissues, we found that the expression of 170 inflammation-related genes changed significantly after HS, and SkQ1 treatment significantly reversed the expression of up to 56.5% of genes. In addition, SkQ1 acted on the proinflammatory and anti-inflammatory genes after HS and altered the expression of 40% of the proinflammatory genes and 58.3% of the anti-inflammatory genes. These results suggest that SkQ1 can effectively reduce the inflammatory response of the heart after HS. The inflammatory response after HS is also manifested in the increased expression of inflammatory factors in blood. Studies have also shown that the excessive production of the proinflammatory factors TNF-α and IL-6 in blood can cause myocardial mitochondrial damage (Liu et al., 2019; Shu et al., 2022). TNF-α can increase the production of mitochondrial ROS, damage mitochondrial structure and integrity, and reduce mitochondrial respiratory function and ATP synthesis, eventually leading to cardiac dysfunction (Medeiros, 2008; Lam et al., 2021). IL-6 regulates peroxisome proliferator-activated receptors-γ coactivator (PGC)-1α, thereby affecting the biogenesis of mitochondria (Eisner et al., 2017). Therefore, the damage to the myocardial mitochondria caused by TNF-α and IL-6 after HS represents a vicious cycle. Hypoxia, oxidative stress, and other factors lead to the increased ROS production and damage to the mitochondrial structure. The damaged mitochondria cause local and systemic inflammation due to the release of MTDs, and the increase in the level of inflammatory factors can further aggravate mitochondrial damage. Our study found that the protein levels of IL-6, MCP-1 and TNF-α in rat blood increased significantly after HS, and SkQ1 could effectively inhibit the increased protein levels, thus providing evidence that SkQ1 can reduce inflammation after HS.

In conclusion, the present study demonstrated that HS causes damage to the rat myocardial mitochondrial structure, increases mtDNA release and ROS contents, and results in the activation of mitochondrial and ROS-related pathways and systemic inflammatory response. By applying SkQ1 for the first time in HS treatment, we report that SkQ1 could reduce inflammation following hemorrhagic shock by protecting myocardial mitochondria, indicating that SkQ1 may be a promising drug for HS treatment.

## Data Availability

The datasets presented in this study can be found in online repositories. The names of the repository/repositories and accession number(s) can be found below: https://www.ncbi.nlm.nih.gov/bioproject/PRJNA880976.
