# Supplementary material for: Mitochondrial antioxidant SkQ1 decreases inflammation following hemorrhagic shock by protecting myocardial mitochondria
Source: Front Physiol. 2022 Nov 16;13:1047909. doi: 10.3389/fphys.2022.1047909 (PMC9709459; doi:10.3389/fphys.2022.1047909)
Supplement: Supplementary file 1 [file DataSheet2.DOCX]

 The link of full original source data is:

<https://www.jianguoyun.com/p/DW2ENyMQ5dj5ChjvsdkEIAA>

The raw data of RNA-seq were uploaded to Sequence Read Archive (SRA) database and the BioProject ID is: PRJNA880976.
