# Supplementary material for: Mitochondrial antioxidant SkQ1 decreases inflammation following hemorrhagic shock by protecting myocardial mitochondria
Source: Front Physiol. 2022 Nov 16;13:1047909. doi: 10.3389/fphys.2022.1047909 (PMC9709459; doi:10.3389/fphys.2022.1047909)
Supplement: Supplementary file 2 [file DataSheet1.docx]

Supplementary Material

1. We first established a 40% fixed-blood-loss hemorrhagic shock rat model and showed the mean artery pressure (MAP) differences between the experimental group and the control group (Supplementary Figure 1, Supplementary Table 1). The blood pressure of the HS rats decreased rapidly during the 20-minute rapid bleeding period and then gradually rose to 68.00±4.297mmHg in 1 hour, but did not return to the normal state as the sham rats. Additionaly, the red blood cell count, hemoglobin content and hematocrit of rats after HS were decreased compared with the sham group (Supplementary Figure 2, Supplementary Table 1).


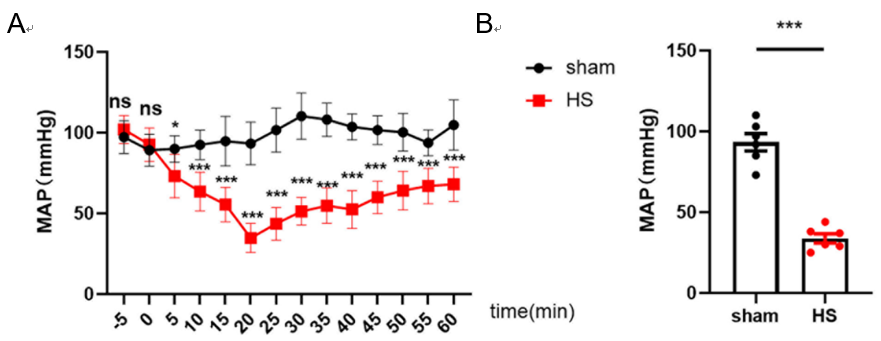


Supplementary Figure 1. MAP decreased during rat blood loss. (A) The MAP was recorded every 5 minutes. 0-20 minutes was the bleeding period, and 20-40 minutes was the observation period after bleeding. (B) MAP statistics at the end of the bleeding period；*n*=6 in each group, ^*^*P* < 0.05, ^***^*P* < 0.001 compared with the sham group.

Supplementary Table 1. MAP data of rats in Sham group and HS group

| Post-surgery duration time  (min) | sham(*n*=6) | | HS(*n*=6) | | *P* Value |
| --- | --- | --- | --- | --- | --- |
|  | Average  (mmHg) | [Standard error](javascript:;) (mmHg) | Average (mmHg) | [Standard error](javascript:;)(mmHg) |  |
| -5 | 97.333 | 4.185 | 102.000 | 3.531 | 0.414 |
| 0 | 89.167 | 4.045 | 92.667 | 4.208 | 0.5621 |
| 5 | 90.000 | 3.357 | 73.167 | 5.504 | 0.0259 |
| 10 | 92.500 | 3.731 | 63.500 | 4.849 | <0.001 |
| 15 | 94.833 | 6.247 | 55.500 | 4.380 | <0.001 |
| 20 | 93.333 | 5.383 | 34.833 | 3.646 | <0.001 |
| 25 | 101.667 | 5.596 | 43.667 | 4.161 | <0.001 |
| 30 | 110.333 | 5.835 | 51.333 | 3.471 | <0.001 |
| 35 | 108.167 | 4.206 | 54.833 | 4.490 | <0.001 |
| 40 | 103.667 | 3.252 | 52.500 | 4.794 | <0.001 |
| 45 | 101.667 | 3.648 | 60.000 | 4.107 | <0.001 |
| 50 | 100.333 | 4.759 | 64.167 | 4.854 | <0.001 |
| 55 | 93.833 | 3.280 | 67.000 | 4.457 | <0.001 |
| 60 | 104.833 | 6.358 | 68.000 | 4.297 | <0.001 |

Supplementary Figure 2. The RBC, HGB and HCT% decreased after HS compared with sham group. Statistical results of the (A) Red blood cell count. (B)Hemoglobin content. (C) Hematocrit. *n*=6 in each group, ^**^*P* < 0.01, ^***^*P* < 0.001 compared with the sham group.

Supplementary Table 2. Blood routine data of rats in Sham and HS group

| type | Sham (*n*=6) | | HS (*n*=6) | | *P* Value |
| --- | --- | --- | --- | --- | --- |
|  | Average | [Standard error](javascript:;) | Average | [Standard error](javascript:;) |  |
| Red blood cell（10^^12^/L） | 6.462 | 0.551 | 2.700 | 0.343 | <0.001 |
| Haemoqlobin（g/L） | 114.3 | 9.196 | 64.57 | 9.512 | 0.003 |
| Haematocrit | 43.33 | 4.759 | 21.00 | 2.633 | 0.002 |

1. Mitochondria volume density was determined with a transparent grid overlaying electron micrograph and the volume density of mitochondria = the number of grid points falling into mitochondria/ the number of total points (Supplementary Figure 3).


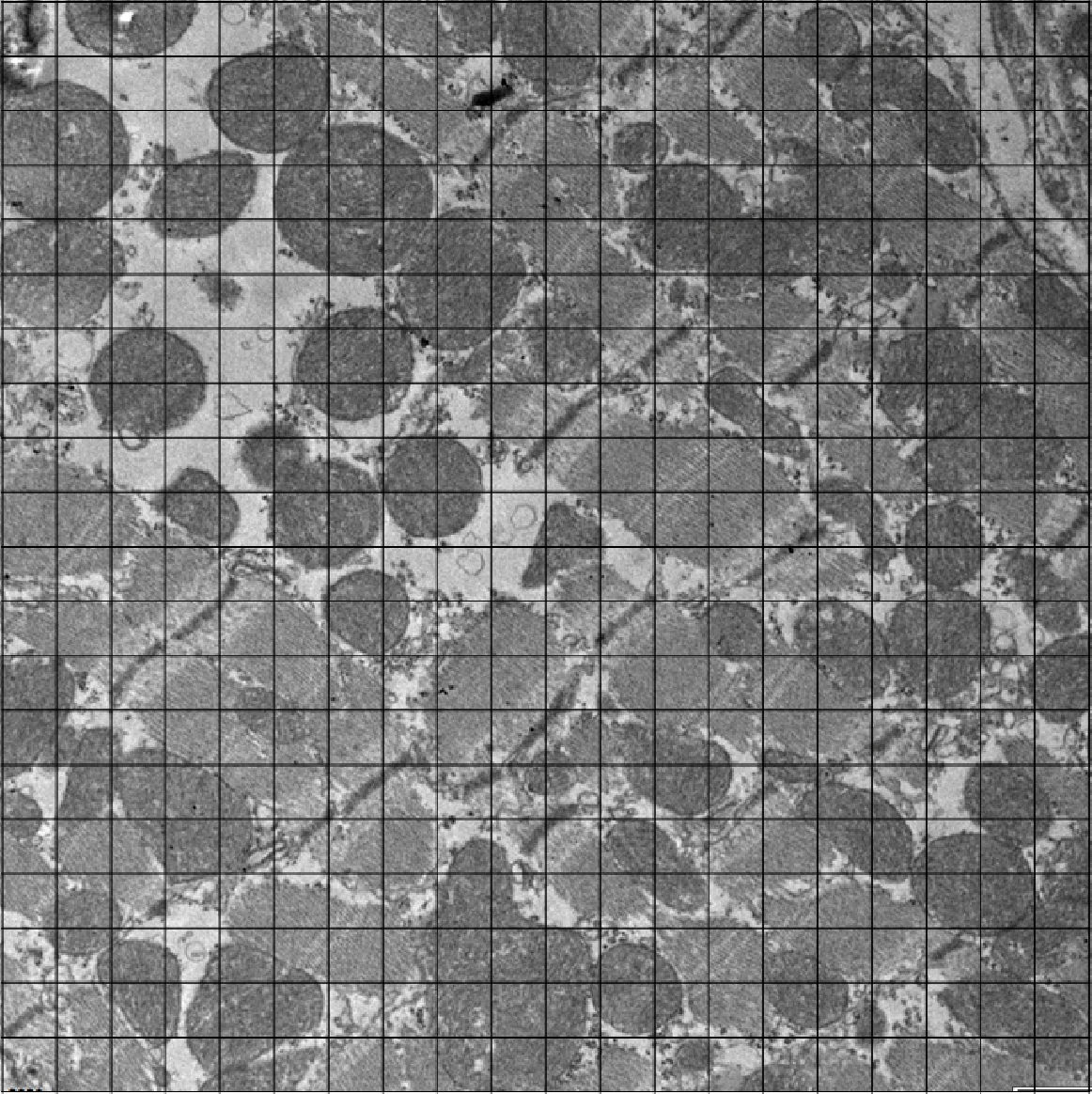


Supplementary Figure 3. Grid-counting method
